# Non-physician clinician provided HIV treatment results in equivalent outcomes as physician-provided care: a meta-analysis

**DOI:** 10.7448/IAS.16.1.18445

**Published:** 2013-07-05

**Authors:** Connor A Emdin, Nicholas J Chong, Peggy E Millson

**Affiliations:** Dalla Lana School of Public Health, University of Toronto, Toronto, ON, Canada

**Keywords:** task shifting, antiretroviral therapy, ART, non-physician, HIV treatment, substitution of physicians

## Abstract

**Introduction:**

A severe healthcare worker shortage in sub-Saharan Africa is inhibiting the expansion of HIV treatment. Task shifting, the transfer of antiretroviral therapy (ART) management and initiation from doctors to nurses and other non-physician clinicians, has been proposed to address this problem. However, many health officials remain wary about implementing task shifting policies due to concerns that non-physicians will provide care inferior to physicians. To determine if non-physician-provided HIV care does result in equivalent outcomes to physician-provided care, a meta-analysis was performed.

**Methods:**

Online databases were searched using a predefined strategy. The results for four primary outcomes were combined using a random effects model with sub-groups of non-physician-managed ART and -initiated ART. TB diagnosis rates, adherence, weight gain and patient satisfaction were summarized qualitatively.

**Results:**

Mortality (*N*=59,666) had similar outcomes for non-physicians and physicians, with a hazard ratio of 1.05 (CI: 0.88–1.26). The increase in CD4 levels at one year, as a difference in means of 2.3 (*N*=17,142, CI: −12.7–17.3), and viral failure at one year, as a risk ratio of 0.89 (*N*=10,344, CI: 0.65–1.23), were similar for physicians and non-physicians. Interestingly, loss to follow-up (LTFU) (*N*=53,435) was reduced for non-physicians with a hazard ratio of 0.72 (CI: 0.56–0.94). TB diagnosis rates, adherence and weight gain were similar for non-physicians and physicians. Patient satisfaction appeared higher for non-physicians in qualitative components of studies and was attributed to non-physicians spending more time with patients as well as providing more holistic care.

**Conclusions:**

Non-physician-provided HIV care results in equivalent outcomes to care provided by physicians and may result in decreased LTFU rates.

## Introduction

In response to the HIV epidemic, countries in sub-Saharan Africa and other low-income regions are attempting to increase access to antiretroviral therapy (ART). The HPTN052 treatment as prevention trial, which demonstrated the ability of ART to prevent HIV transmission, has provided further impetus for nations to provide universal ART access [1]. However, the severe healthcare worker shortage affecting many of these countries is inhibiting the expansion of therapy. Malawi, for example, has only 2 physicians for every 100,000 individuals, compared to 247 per 100,000 in the United States [2]. Task shifting, the transfer of responsibilities to different cadres of healthcare workers, has been proposed to address this problem. The most widely proposed form is the gradual transfer of ART management and initiation from doctors to nurses and other non-physician clinicians. The World Health Organization has proposed that this and other forms of task shifting be implemented across sub-Saharan Africa to increase the availability of antiretrovirals (ARVs) [3].

There is a large body of research evaluating the effects of task shifting policies with regard to access, outcomes and cost. Many field reports have described using task shifting policies, particularly nurse-initiated ART, as critical to increasing access to HIV treatment [4]. However, many of these reports suffer from confounding factors, including substantial external funding, intensive training of staff by overseas NGOs and innovative programmes, factors not present in most resource-limited health systems [4]. A recent systematic review concluded that while task shifting policies could improve access to ARVs, they must be implemented with training and support and should be considered as a single tool to be used with other health system strengthening policies [5]. Recent studies have also concluded that nurse-managed care results in lower costs than physician-managed care and that task shifting policies in general have good outcomes [3].

Considering that task shifting policies appear to be able to increase access to HIV treatment with a reduced cost, it would be reasonable to think that there would be widespread implementation of task shifting in low-income countries. However, formal implementation of nurse-managed and -initiated HIV treatment remains limited. While Rwanda and South Africa, among other countries, have begun to phase in nurse-initiated ART, many countries in sub-Saharan Africa limit prescription of ARVs to physicians [6]. There remain significant barriers to implementation of task shifting policies. Most prominently, many policy makers and governmental ministers have limited knowledge of task shifting policies and question the ability of task shifting to result in equal quality of care. Until task shifting is formalized within a governmental framework, the ability of training programmes for non-physicians to be implemented remains limited [6].

In order for policy makers to provide evidence-based guidelines for task shifting policies, the highly heterogeneous literature must be evaluated and pooled. Although recent systematic reviews have qualitatively evaluated the impact task shifting policies have on access to ART [5] and summarized the use of task shifting in Africa generally, [3] none have quantitatively summarized the literature using a meta-analysis. This systematic review aims to fill this void and assess the quality of care provided by non-physician-initiated and -managed HIV treatment.

## Methods

This systematic review was performed using the meta-analysis of observational studies in pidemiology (MOOSE) statement as a guideline. This systematic review was also registered with the International Prospective Register of Systematic Reviews (ID: CRD42012001956) prior to its initiation, with the search strategy described there.

### Inclusion criteria

Task shifting was defined for this review as non-physician managed and initiated ART. Non-physician clinicians were considered to be nurses, clinical nurses, registered nurses, nurse practitioners, nurse specialists as well as specialized non-physician clinicians such as clinical officers. Trainees and community health workers were excluded. Four separate types of studies were eligible for inclusion in the meta-analysis: randomized controlled trials, non-randomized controlled trials, cohort studies and case-control studies. Cross-sectional studies were eligible for narrative review but not included in the meta-analysis. Studies which measured either non-physician-initiated or -managed ART were eligible. Non-physician-initiated ART refers to non-physicians both initiating and managing ART, while non-physician-managed ART refers to non-physicians refilling prescriptions and managing treatment after diagnosis and initial prescription by a physician. Studies which measured either adults or children (0–18 years) were also eligible for inclusion. No language or age restrictions were applied.

### Search strategy

The following databases were searched until August 2012: PubMed, EMBASE, CINAHL, Social Science Citation Index, Arts and Humanities Index, Cochrane Controlled Trials Registry as well as the abstract databases from the International AIDS Society Conferences, the Conferences on Retroviruses and Opportunistic Infections and the conferences of the International Society of Sexually Transmitted Diseases Research. Bibliographies of relevant studies were manually searched. A grey literature search was also conducted.

The search strategy combined three themes: HIV/AIDS, ART and task shifting. The detailed strategy is described in Supplementary File 3. A variety of synonyms were used for each theme and both keywords and MeSH headings were used. No keywords were used for outcomes or quality of care, as relevant studies may not have contained these keywords. Instead, papers were reviewed manually.

### Study selection and data extraction

Two reviewers (CE and NC) performed the search independently, screening studies by title and abstract. Those studies identified as being possibly relevant to ART task shifting were assessed using the full text by two reviewers (CE and NC). Once the full-text screen was performed, differences between the eligible studies were resolved by consensus. Excluded studies with the reasons for exclusions were recorded and provided. Information was extracted independently and in duplicate using a pre-created form. The primary outcomes were death, loss to follow-up (LTFU), CD4 increase at one year and virological failure at one year. Secondary outcomes were patients requiring change of ART prescription, change in weight, documented medical or prescription failures, prevalence of opportunistic infections, adherence and patient satisfaction. Performance indicators, including the ability to properly stage HIV infection, prescribe ART and document treatment, were also evaluated.

### Quality assessment

Quality of individual studies was assessed using a series of predefined measures. Randomized controlled trials and controlled before and after studies were assessed using eight measures: calculation of power, description of eligibility, baseline assessment, incomplete outcome data, selective outcome reporting, clear description of roles of nurses, measurement of implementation of intervention and discussion of confounding factors. Cohort studies were assessed through the same measures with the removal of calculation of power. No case-control studies were eligible for inclusion, however, if one had been identified, it would have been assessed through the same seven measures as a cohort study. Cross-sectional studies were assessed with the same measures with the additional removal of baseline assessment. Studies which described and reported each measure were marked as having fulfilled that assessment. If the study did not report the measure, it was marked as not having fulfilled the quality assessment. For selective outcome reporting, protocols published prior to the trial being performed were used to determine if select outcomes had been excluded. As a result, studies which did not have previously published protocols were assessed as being unclear for selective outcome reporting.

### Data analysis

The software RevMan 5.0, provided by the Cochrane collaboration, was used for all statistical calculations. A meta-analysis was applied for each sub-group of non-physician-managed ART and -initiated ART using a DerSimonian–Laird random effects model, which was chosen over a fixed effects model as there was heterogeneity between studies. *I* ^2^ was calculated for each sub-group as a measure of between-study heterogeneity. For all outcomes, the generic inverse variance method was used to report pooled summary statistics. For outcomes reported as hazard ratios (mortality and LTFU), Parmar's methods were used to extract hazard ratios and standard errors from reported confidence intervals and were also used to estimate hazard ratios for studies which reported total mortality at discrete time points [7]. An increase in CD4 levels at one year combined was reported as a continuous outcome while rates of viral failure at one year, considered to be patients with >400 copies/mL, was reported as a risk ratio. Funnel plot asymmetry was used to detect possible publication bias.

## Results

**Figure 1:**
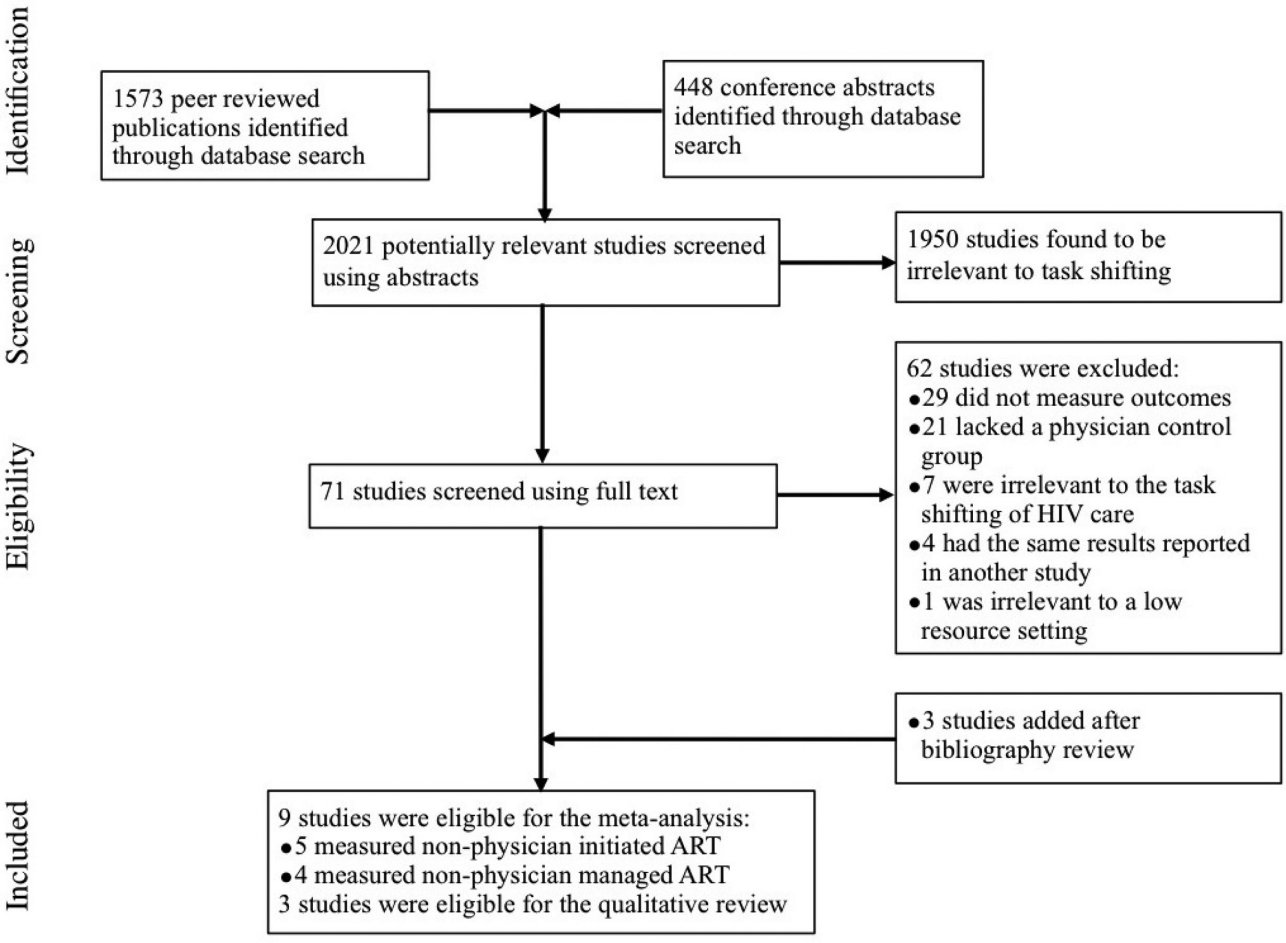
Flow diagram of studies selected for systematic review and meta-analysis of non-physician-provided HIV care.

Of the 2021 studies screened by title and abstract, 71 were screened using the full text (Figure 1). Of those, 62 were excluded. The reasons for each study's exclusion are provided in Supplementary File 3. In summary, 29 did not measure outcomes, 21 lacked a control group, 7 were irrelevant to the task shifting of HIV care to non-physician clinicians, 4 had results reported in another study and 1 was irrelevant to low-resource settings. Three studies were located by reviewing the bibliography of relevant studies. Of the remaining 12 studies, 9 studies were eligible for the meta-analysis while 3 studies were only eligible for the qualitative review.

### Study characteristics

Nine studies were included in the meta-analysis (Table 1). Of those, four measured ART managed but not prescribed by nurses [8–11], three measured ART prescribed by nurses [12–14], one measured ART prescribed by both nurses and health officers [15] and one measured ART prescribed by a mid-level care provider [16]. One study was a cluster randomized controlled trial [17], one was an individual randomized trial [9], one study was a non-randomized controlled trial [11] and six were retrospective cohort studies [8, 10, 12, 13, 15, 16]. All nine studies measured mortality while eight measured LTFU, five measured CD4 changes and five measured viral load changes. Five of the studies were situated in South Africa [8, 10, 13, 14] , one was conducted in Mozambique [16], one in Ethiopia [15], one in Lesotho [12] and one in Swaziland [11].

**Table 1:** Characteristics of studies included in the meta-analysis and qualitative review

Study	Study design	Subjects	Location and date	Type and role of non-physicians	Participant population	Outcomes measured
Meta-analysis						
Assefa, 2011 [15]	Retrospective cohort among 30 hospitals (doctor-led) and 25 health centres (nurse-led)	37,475	Ethiopia, Sept. 2006 to Mar. 2009	Health officer and nurse-initiated ART	All patients who started between Sept. 2006 and Aug. 2008	Mortality, LTFU, CD4, patient attitudes
Brennan, 2011 [8]	Retrospective matched cohort analysis of a clinic (doctor-led) and a down-referral clinic (nurse-led)	2772	Johannesburg, SA, Feb. 2008 to Dec. 2009	Nurse-managed ART	>18 years, on first line ART, had all data needed for matching	Mortality, LTFU, CD4, viral load rebound, patient satisfaction
Bedelu, 2007 [13]	Retrospective cohort of 1 hospital and 12 clinics	1025	Lusikiski, SA, Jan. 2004 to Jul. 2006	Nurse-initiated ART	All patients who completed 12 months of treatment by Jul. 2006	Mortality, LTFU, CD4, viral load
Fairall, 2011 [14]	Cluster randomized controlled trial of 31 clinics	6321	Free state province SA, Jul. 2007 to Dec. 2010	Nurse-initiated ART	Patients >18 years of age, on ART for >6 months (patients were reinitiated)	Mortality, viral load, CD4, change in ART, TB diagnosis
Humphreys, 2010 [11]	Non-randomized controlled trial of 15 nurse-led ART clinics and 11 primary care clinics	474	Lubombo, Swaziland Jan. 2007 to Nov. 2007	Nurse-managed ART	>14 years of age with CD4 >100	Mortality, LTFU, CD4, weight, patient satisfaction
Labhardt, 2012 [12]	Retrospective cohort study of 2 hospitals and 12 health centres	3733	Lesotho, 2008 to 2011	Nurse-initiated ART	>16 years of age	Mortality, LTFU
Pienaar, 2008 [10]	Retrospective cohort study of 4 doctor-managed clinics and 1 task shifting clinic	564	Western Cape, SA, Jul. 2004 to Jun. 2005	Nurse-managed ART	>18 years of age, ART naive	Mortality, LTFU, CD4, viral failure, adherence
Sanne, 2010 [9]	Randomized controlled trial at 2 clinics	812	South Africa, Feb. 2005 to Nov. 2007	Nurse-managed ART	>16 years of age, CD4<350 or AIDS-defining illness	Mortality, LTFU, viral and treatment failure
Sherr, 2010 [16]	Retrospective cohort at 2 public HIV clinics	5892	Mozambique, Jul. 2004 to Oct. 2007	Mid-level care provider-initiated ART	>15 years of age	Mortality, LTFU, CD4, optimal adherence
Qualitative review						
Boyer, 2011 [18]	Cross-sectional study evaluating factors associated with adherence in 27 health centres	2381	Cameroon, Sept. 2006 to Mar. 2007	Nurse-managed ART	>21 years of age, receiving ART	Adherence, unplanned treatment interruptions
Monyatsi, 2011 [17]	Cross-sectional survey comparing the performance of physicians to nurses	197 providers	Gaborone, Botswana Jan.–Mar. 2009	Nurse-initiated ART	Paediatric patients <16 years of age, on ART for one year	Appropriate documentation for ight variables
Vasan, 2009 [19]	Cross-sectional evaluation comparing agreement between non-physicians and physicians	521	Uganda, Jul. to Sept. 2006	Nurse- and clinical officer-initiated ART	>18 years of age, not started on ART	Agreement whether to initiate ART, therapy prescribed

Three cross-sectional studies were included in the narrative review. One was conducted in Botswana, measuring nurse-initiated ART [17], one in Cameroon, measuring nurse-managed ART [18] and one in Uganda measuring nurse- and clinical officer-initiated ART [19]. Two evaluations compared the performance of non-physicians to physicians [17, 19], while one evaluated factor associated with adherence to therapy [18].

### Study quality

The methodological quality of the studies varied significantly (Supplementary File 1). Only two of the nine studies in the meta-analysis were randomized controlled trials with a third study being a non-randomized controlled trial. All controlled trials had at least six of the eight quality factors. The remaining five cohort studies were of lower methodological quality, with two cohort studies having only three of the seven factors. The remaining four had at least four of the factors. The most common error among all studies was a lack of an evaluation of the degree to which nurse-managed and -initiated care was actually being implemented within the clinic. The STRETCH randomized controlled trial was one of the few studies which monitored the implementation of nurse-initiated ART [14]. Within nurse-initiated clinics, only 26% of ART initiations were by a nurse. It is thus unclear whether, in studies which did not monitor the implementation of nurse-initiated ART, a large majority of ART initiations were actually by physicians. The three cross-sectional studies included in the qualitative review were also of varying quality, with one study having only two of six factors while the two remaining studies had three of five. It was also unclear for the cohort and cross-sectional studies whether there was selective outcome reporting, as protocols for retrospective studies were not published prior to the study being conducted. As only two studies measured all four of the primary outcomes (both randomized controlled trials), selective outcome reporting remains a significant concern for the nine observational studies included in this systematic review [8, 9].

### Primary outcomes

**Figure 2:**
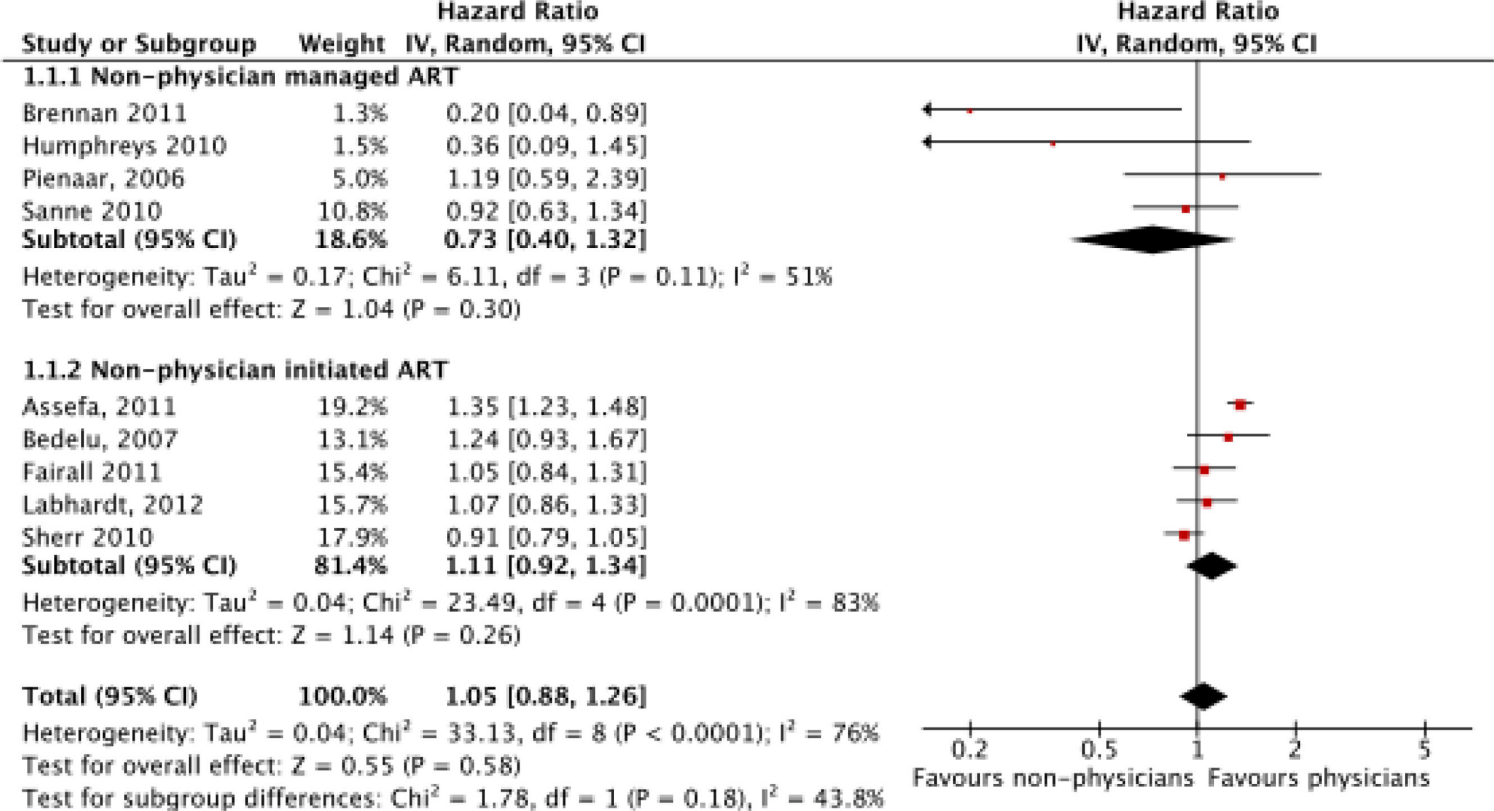
Meta-analysis of mortality (*N*=59,666), separated by sub-group analysis of nurse-managed and nurse-initiated ART.

**Figure 3:**
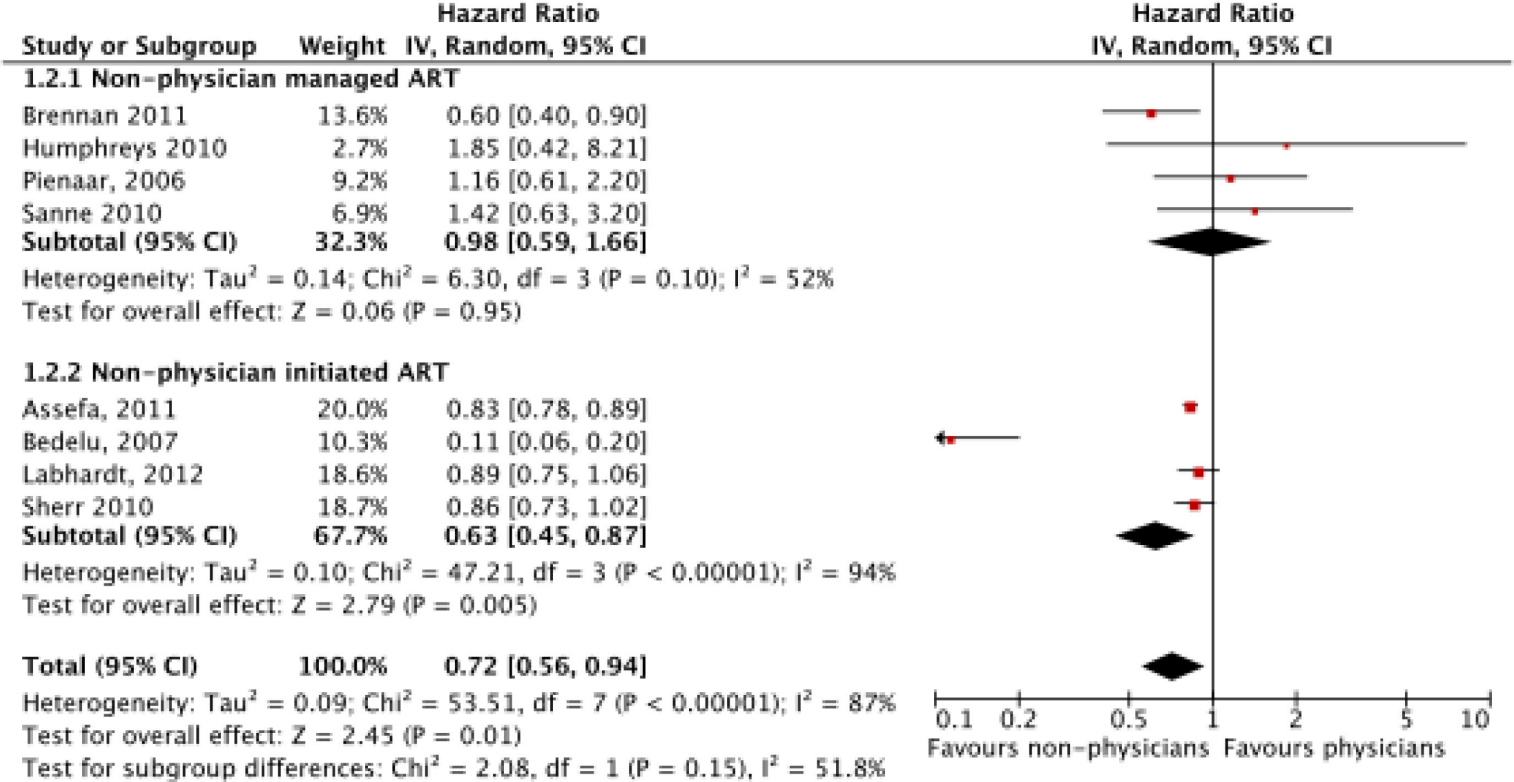
Meta-analysis of loss to follow-up (LTFU) (*N*=53,435), separated by sub-group analysis of nurse-managed and nurse-initiated ART.

Funnel plots were produced for mortality and LTFU to determine if there was publication bias (Supplementary File 2). While there were too few studies to apply a quantitative test for bias, visual inspection of the plot did not indicate significant bias. There was significant heterogeneity between studies with *I* ^2^=76% (*p*<0.0001) for mortality and *I* ^2^=87% (*p*<0.00001) for LTFU. Consequently, a random effects model was used for analysis. Meta-analysis of mortality (*N*=59,666) found no significant difference between non-physicians and physicians with a hazard ratio of 1.04 (CI=0.84–1.28, *p*=0.72) (Figure 2). In a sub-group analysis, there was no significant difference between non-physicians and physicians for non-physician-managed ART (*p*=0.30) or -initiated ART (*p*=0.31). However, non-physicians had reduced LTFU (*N*=53,435), with a hazard ratio of 0.72 (CI=0.56–0.94, *p*=0.01) (Figure 3). This effect was primarily driven by the non-physician-initiated ART group which had a ratio of 0.63 (CI=0.45–0.87), while the non-physician-managed ART subgroup had a hazard ratio of 0.98 (CI=0.59–1.66). When Bedelu (2007) [13] is removed in a sensitivity analysis, the effect for both non-physician-initiated ART sub-group (HR=0.84, *p*<0.00001) and combined sub-groups (HR=0.85, *p*<0.0001) remains.

**Figure 4:**
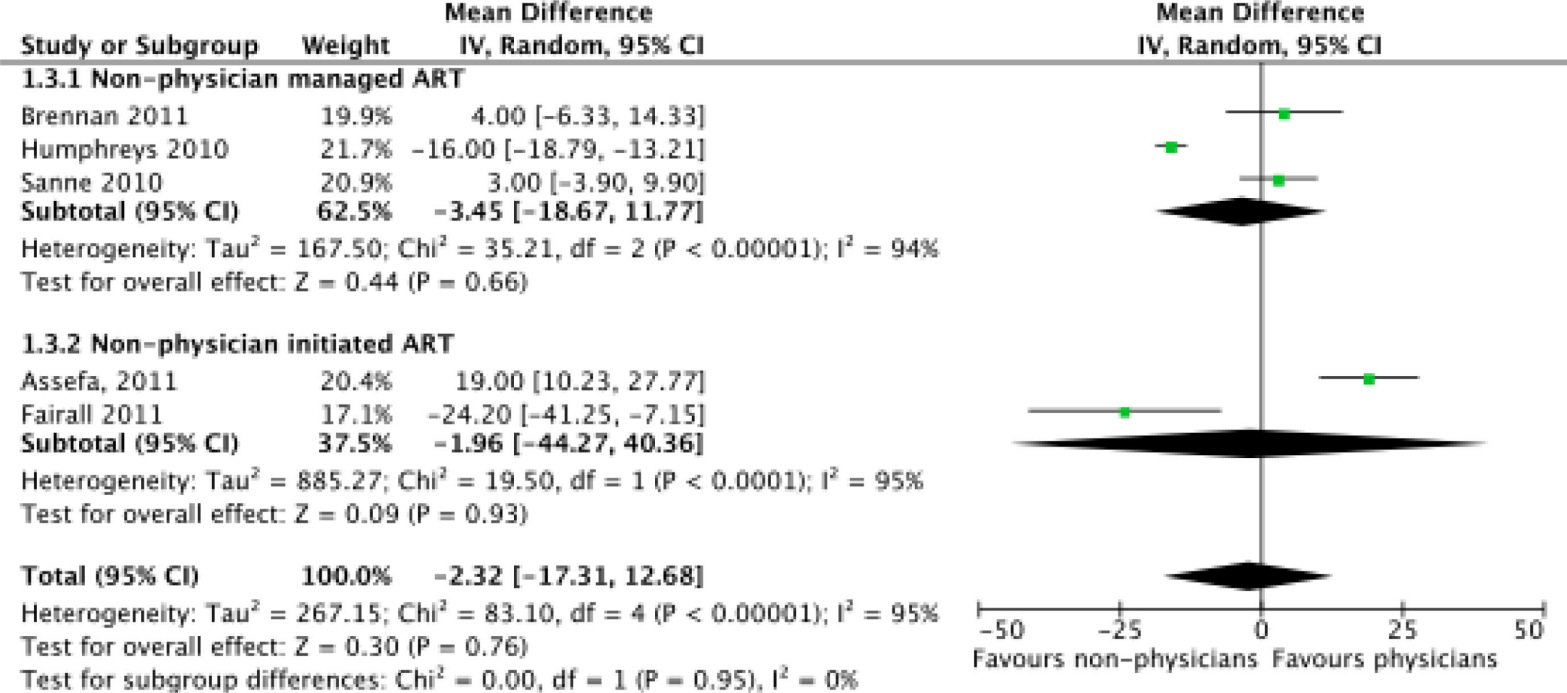
Meta-analysis of increase in CD4 levels at one year (*N*=17,142), separated by sub-group analysis of nurse-managed and nurse-initiated ART.

**Figure 5:**
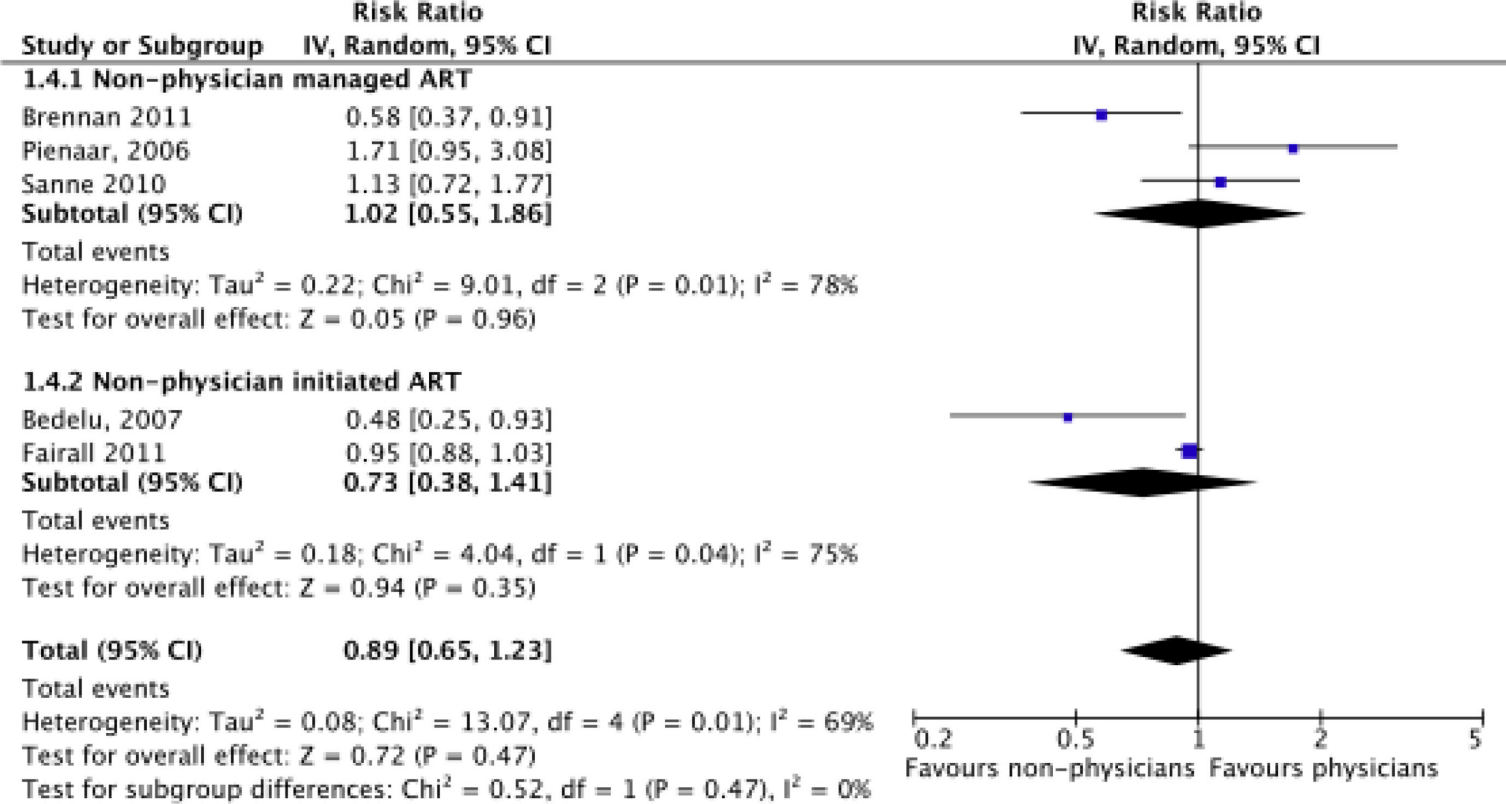
Meta-analysis of rates of viral failure at one year (*N*=10,334), separated by sub-group analysis of nurse-managed and nurse-initiated ART. *Viral failure is defined as >1000/mL rather than >400/mL for Sanne, 2010 [9].

Meta-analysis of CD4 level increase at one year (*N*=17,142) found no evidence of a significant difference between physicians and non-physicians, with a mean difference of −2.32 favouring non-physicians (CI=−17.31 to 12.68, *p=*0.76) (Figure 4). Neither sub-group was found to have a significant difference between types of clinicians although the small number of trials reduced the power of the analysis. The number of patients in viral failure at the end of one year (*N*=10,344), defined as having >400 units/mL of blood, was also found not to be significant between non-physicians and physicians (risk ratio=0.89, CI=0.65–1.23, *p*=0.47) (Figure 5). Significant differences in rates of viral failure were also not found in sub-group analysis.

### Secondary outcomes

Eight studies reported secondary outcomes (Table 2). In two randomized controlled trials evaluating non-physician-provided HIV care, the rates of TB diagnosis were equivalent between non-physicians and physicians [9, 14]. The rates of toxicity failure were also found to be similar between nurses and physicians in one randomized trial in South Africa [9]. Two studies compared patient's weight change following nurse provided HIV care and physician provided care. A non-randomized trial in Swaziland found similar levels of weight increase when treated by physicians or non-physicians [11], while a randomized trial in South Africa found that patients treated by nurses had a significantly greater weight increase at one year by 0.77 kg [9]. Rates of CD4 testing were found to be similar between physicians and non-physicians in a cohort study in Mozambique [16], while a cohort study in South Africa found that patients treated by nurses were significantly more likely to have had CD4 levels completed than patients treated by physicians [13]. The only randomized trial which evaluated change in ART therapy found that nurses were more likely to change therapy with a risk ratio of 2.99 [9].

**Table 2:** Summary of studies that evaluated secondary outcomes

Study	Type and role of non-physician	Outcomes and result
Assefa, 2011 [15]	Health officer and nurse-initiated ART	Satisfaction: Patients were more comfortable with nurses and health officers who were friendlier and more supportive.
Boyer, 2011 [18]	Nurse-managed ART	Adherence: Patients going to hospitals with task shifting had non-significant decreased likelihood of non-adherence: OR=0.59, CI=(0.26–1.33). Treatment interruptions: Patients going to hospitals with task shifting had a non-significant increased likelihood of treatment interruption OR=01.29, CI=(0.49–1.38).
Bedelu, 2007 [13]	Nurse-initiated ART	CD4 testing: Patients getting nurse-initiated treatment at clinics were significantly more likely to have CD4 levels done than patients with physician-initiated ART (*p*<0.001).
Fairall, 2011 [14]	Nurse-initiated ART	Change in weight: Patients had a greater weight increase for nurses (1.3 kg) than for physicians (0.47 kg). Δ=0.77 kg CI=(0.20,1.34). TB diagnosis: There was no significant difference in likelihood of TB diagnosis of nurses and physicians. RR=1.11, CI= (0.86, 1.43). Change of ART drugs: Nurses were more likely to change antiretroviral therapy. RR=2.99, CI=(2.21,4.02).
Humphreys, 2010 [11]	Nurse-managed ART	Change in weight: There was no significant difference between weight gain for nurses (1.09 kg) than for physicians (1.04 kg). Satisfaction: Patients had a non-significant preference for nurse-managed care than physician-managed. RR=1.25, (CI=0.97–1.61).
Pienaar, 2008 [10]	Nurse-managed ART	Satisfaction: Patients preferred to see nurses over doctors. Nurses were considered to be more holistic.
Sanne, 2010 [9]	Nurse-managed ART	Toxicity failure: There was no significant difference in likelihood of toxicity failure for nurses than physicians. RR=1.04, CI=(0.74–1.45). TB diagnosis: There was no significant difference in likelihood of a diagnosis of TB for nurses than physicians. RR=0.92, CI=(0.55–1.53).
Sherr, 2010 [16]	Nurse- and clinical officer-initiated ART	Adherence: Patients being cared for by non-physicians had higher rates of adherence than physicians. RR=1.05 CI=(1.02–1.09). CD4 Testing: There was no significant difference between CD4 testing by non-physicians and physicians at one year of ART. RR=1.12 (CI=0.96–1.31).

Satisfaction rates were generally found to be better for non-physicians than physicians. Two qualitative components of cohort studies found that patients expressed a preference for nurses over physicians, who were seen as friendlier, more supportive and providing more holistic care [10, 15]. The single quantitative measurement of satisfaction found that patients had a non-significant preference for nurse-managed ART with a risk ratio of 1.25 [11].

A cross-sectional study in Cameroon evaluated which factors were associated with non-adherence and treatment interruptions to ART [18]. Attending a healthcare facility with a policy of task shifting HIV care to nurses was associated with a decreased risk of non-adherence (OR=0.59, 0.26–1.33) but with a higher risk of treatment interruptions (OR=1.29, 0.49–3.38). Neither association was statistically significant. In a cohort study in Mozambique, however, patients cared for by non-physicians were found to have significantly higher rates of adherence [15].

### Performance indicators

Two cross-sectional studies compared non-physicians to physicians on performance indicators. A study in Botswana compared the ability of nurses and physicians to properly document eight variables including WHO staging and dosing of therapy for paediatric HIV patients [17]. Nurses correctly documented 96% of the time compared to 94.9% for physicians, demonstrating non-inferiority of nurse performance. Nurses also had higher rates of proper social history documentation. A study in Uganda compared the agreement between nurses, clinical officers and physicians on whether to initiate ART and the form of therapy prescribed [19]. Between nurses and physicians and clinical officers and physicians, there was 95% and 95.9% agreement, respectively, on the recommendation of ART. With regard to WHO stage, there was 96.6% and 94.2% agreement for nurses and clinical officers, respectively.

## Discussion

Our meta-analysis and systematic review found that non-physician-provided care resulted in equivalent outcomes to physician-provided treatment. This is consistent with the two randomized controlled trials included in this meta-analysis [9, 14], as well as other non-controlled cohort studies which have reported good outcomes for nurse-provided care [20, 21]. Non-physician-initiated ART was found to have reduced LTFU compared to physician provided care, while non-physician-managed ART was found to have no effect. This is likely due to a difference in the design of non-physician-managed and non-physician-initiated studies. The comparison group in studies measuring non-physician-initiated ART were entire clinics which used non-physician-initiated and -managed care, while studies measuring non-physician-managed ART used clinics which had both nurses and physicians simultaneously providing care as their comparison group. Decentralization of care is likely responsible for the reduced LTFU rate. Treatment at primary care clinics closer to individuals’ communities may reduce waiting times and allow patients to take less time off work, increasing the likelihood of patients remaining in care. As studies of non-physician-initiated ART tended to have decentralization more than managed ART studies, a reduced LTFU rate was found for non-physician-initiated ART. When performing a separate sub-group analysis, splitting studies which used separate clinics for non-physicians and physicians against those in which they worked together, the effect is pronounced with separate clinics having a mortality HR=0.64 (CI: 0.44–0.93) while the same clinic has HR=0.94 (CI: 0.65–1.36). Thus, the decentralization often associated with task shifting to non-physicians reduces LTFU.

Many of the strengths of this review can also be considered weaknesses. No restrictions were put on publication status and both peer-reviewed papers as well as conference abstracts were accepted for inclusion. This was done to reduce the risk of publication bias and include as many studies as possible. Two abstracts [10, 12] were included in the meta-analysis as published studies were found which described the outcomes of other eligible abstracts. The lack of peer review for these abstracts may mean that issues with their methodology were not uncovered. However, exclusion of these studies did not have a significant effect on the calculated summary mortality hazard ratio, changing from 1.05 (CI: 0.88–1.26) to 1.03 (CI: 0.82–1.28).

A second potential pitfall with this meta-analysis relates to the use of Parmar's methods for extracting hazard ratios from summary statistics [7]. While the use of Parmar's methods allowed us to include a variety of studies reporting mortality data in various forms, the approximations used in the methods may have introduced error into the calculated summary statistics for certain studies. For example, a cohort study [15] reported net mortality rates at only four separate time points while another cohort study [13] reported only total mortality at the final time point. The extracted time to death hazard ratios for these two studies will be an estimate of the actual time to death ratio, as Parmar's method assumes a constant hazard rate between time points. However, exclusion of these smaller studies would introduce a significant risk of publication bias. When a separate subgroup analysis is performed with these studies removed, the overall hazard ratio for morality remains similar, changing to 0.96 (CI=0.82–1.12).

A strength of our meta-analysis is the inclusion of both randomized trials and observational studies. Randomized trials typically have higher internal validity than observational studies. However, they may lack applicability to the health systems typically found in sub-Saharan Africa [22]. This can be seen in the CIPRA randomized trial in South Africa, included in this meta-analysis, which compared nurse-managed ART to physician-managed treatment in 808 patients. In addition to training in the use of ART, nurses and physicians were provided with didactic clinical management as well as telephone support from a clinical safety team, interventions which would not be present in most low-resource settings. The inclusion of observational studies, which typically occur under conditions similar to the external health system, should balance this weakness of randomized trials. Other confounding factors, including the presence of overseas non-governmental organization funding, talented leadership and intensive training, were found in both observational and randomized studies and may hinder the applicability of their findings to an external health system [4].

The quality of the included studies, analyzed through a set of assessments defined *a priori*, varied significantly. The two included randomized controlled trials were of higher quality than the included cohort studies, although they did not differ significantly in their findings. The most troubling factor among most studies was a lack of an assessment to a degree to which nurses actually initiated and managed ART. In the STRETCH trial, only 26% of initial prescriptions of ARVs in the nurse-led cohort were actually prescribed by nurses [14]. This raises the question of whether studies that compared non-physician-led clinics to physician led-clinics inadvertently included large numbers of patients treated by physicians within non-physician-led cohorts.

This meta-analysis supports the general trend in sub-Saharan Africa towards the provision of ART by nurses and other non-physicians. Many countries, including South Africa, Lesotho and Malawi have implemented national policies allowing the prescription of ARVs by nurses [23]. However, many countries which suffer from poor access to ART, including Mozambique, Tanzania and the Democratic Republic of Congo, continue to restrict prescription of ARVs to physicians [24]. It has been argued that this lack of implementation of task shifting policies is a form of unnecessary rationing of medical care and is unethical [6]. However, significant barriers remain before task shifting policies can be implemented throughout sub-Saharan Africa. There remains unwarranted resistance among health officials to putting in place formal regulatory frameworks for nurses to prescribe ART [6]. There is a significant cost to implementing novel training programmes for non-physicians and there is concern among nurses about taking on additional tasks for which they will not be remunerated [15]. In addition, task shifting of ART to overworked non-physicians may be ineffective at increasing access to ART if it is not combined with the shifting of existing tasks from non-physicians to lower level cadres [5]. Eventually, shifting of ART management tasks may go beyond non-physician clinicians to community health workers and even patients themselves. Finally, there is the ever-present concern that task shifting of ART to non-physicians will deplete already limited resources for general health services [15]. In this regard, task shifting can be considered a short-term solution to be used in combination with broader measures to strengthen healthcare systems, including increased funding, training and evidence-based policy making.

These barriers suggest areas for future research. First, research should be conducted into the ability of various combinations of task shifting to non-physicians and lower level cadres to increase access to HIV treatment. Second, the cost effectiveness of task shifting to non-physicians should be examined. A recent cohort study, conducted in South Africa, concluded that down-referral to a nurse-managed clinic was 11% less expensive than physician provided care due to nurses ordering more expensive drugs. However, if down-referral was implemented throughout the South African healthcare system, even its relatively moderate reduction in cost could provide for millions of dollars in savings at an aggregate level [25]. Comprehensive guidelines for the prescription of ART by nurses could prevent discrepancies between physician and non-physician prescription patterns, further increasing potential savings. Third, training programmes for nurses and other non-physicians need to be evaluated on their ability to prepare non-physicians to independently prescribe ARVs. While the ideal programme should be relatively short and incur a low cost, these requirements may conflict with training non-physicians to provide quality care. Supervision after training is also necessary to improve patient care and ensure that knowledge from training programmes can be translated into practice. Finally, additional barriers to implementing task shifting policies on national levels need to be identified. Researchers should consider sharing the results of these studies with advocacy and policy organizations to increase the level of awareness of task shifting among health officials.

## Conclusions

This meta-analysis of the result of a combined 59,666 subjects found that non-physician-provided HIV treatment results in the same outcomes as traditional physician-provided treatment and may result in reduced LTFU rates. Patients were more likely to be satisfied by care provided by non-physicians, which was attributed to the perception that nurses provided more holistic care and that nurse-led clinics were closer to patients’ homes. These results support the current expansion of task shifting policies in sub-Saharan Africa and suggest that countries that have not implemented such policies may be unnecessarily rationing their care. Future research should be conducted into evaluating training programmes for non-physicians as well as identifying barriers that are inhibiting the expansion of task shifting policies.
